# Use of Germination to Enhance Resveratrol Content and Its Anti-Inflammatory Activity in Lipopolysaccharide-Stimulated RAW264.7 Cells

**DOI:** 10.3390/molecules28134898

**Published:** 2023-06-21

**Authors:** Chaiwat Monmai, Jin-Suk Kim, So-Hyeon Baek

**Affiliations:** Department of Agricultural Life Science, Sunchon National University, Suncheon 59722, Republic of Korea; bbuayy@gmail.com (C.M.); kimjs6911@naver.com (J.-S.K.)

**Keywords:** transgenic rice, resveratrol, piceid, anti-inflammation, germination, nuclear factor kappa B, mitogen-activated protein kinase

## Abstract

Inflammation is triggered by a variety of danger signals and is now a worldwide concern. Resveratrol, a natural nonflavonoid polyphenol found in naturally consumed plants and foods, has a wide spectrum of bioactive potency. We successfully generated resveratrol-enriched rice by introducing the resveratrol biosynthesis gene into Dongjin rice. In this study, resveratrol- and piceid-enriched rice (DJ526) was investigated for its anti-inflammatory activity in lipopolysaccharide (LPS)-stimulated RAW264.7 cells compared to normal rice (DJ). In addition, the 5-day-old germinated DJ526 (DJ526_5) was tested for its anti-inflammatory effects. The piceid and resveratrol amounts increased in DJ526_5 by germination. Treatment of LPS-stimulated RAW264.7 cells with resveratrol-enriched rice seed extracts (DJ526_0 and DJ526_5) significantly decreased the production of nitric oxide (NO) and the inflammatory mediator prostaglandin E2 (PGE_2_), downregulated proinflammatory gene expression, and inhibited nuclear factor kappa B (NF-κB) p65, p38 mitogen-activated protein kinase, and extracellular signal-regulated kinase 1/2 (ERK 1/2) phosphorylation. These findings demonstrated the anti-inflammatory mechanisms of resveratrol-enriched rice in LPS-stimulated RAW264.7 cells. Furthermore, resveratrol-enriched rice could be a potential source of anti-inflammatory agents.

## 1. Introduction

Inflammation is a complex set of interactions between soluble factors and cells that can occur in any tissue in response to a traumatic, infectious, postischemic, toxic, or autoimmune injury [1]. It is a protective biological response to harmful stimuli, pathogens, or irritants in vascular tissues that attempts to eliminate infectious agents [2]. Immune protection against pathogens also involves the activation of various signaling pathways, including the mitogen-activated protein kinase (MAPK) and nuclear factor kappa B (NF-κB) pathways [3]. During infection, the host produces several proinflammatory cytokines that are implicated in disease pathogenesis [4]. Macrophages play essential roles in inflammation [5]. Interleukin-6 (IL-6) is a multifunctional cytokine that promotes B-cell differentiation [6] and has a wide range of biological activities in immune regulation, hematopoiesis, inflammation, and oncogenesis [7]. Furthermore, IL-1β and tumor necrosis factor-alpha (TNF-α) also play crucial roles in inflammation and osteolysis [8]. IL-1β, a proprotein produced by activated macrophages, is an important mediator of the inflammatory response and is involved in a variety of cellular activities, such as cell proliferation, differentiation, and apoptosis [9].

Cytokines are necessary, but their constitutive overproduction or prolonged or chronic inflammation is often involved in a variety of diseases, such as IL-17-driven inflammatory responses in human colon adenocarcinomas [10]. Inflammation promotes tumor development and increases the prevalence of chronic inflammatory lung diseases [11], and proinflammatory factors are associated with type 2 diabetes [12]. Therefore, inhibiting the expression and production of powerful mediators such as IL-6 and TNF-α by anti-inflammatory components could be a preventive or therapeutic target and could be used to develop anti-inflammatory agents for health promotion and disease prevention [13].

Resveratrol (3,5,4′-trihydroxy-trans-stilbene) is a stilbenoid or diarylethene polyphenol found in a variety of plants and foods, including peanuts [14], mulberries [15], grapes [16], red wine [17], and other plants [18,19,20]. Several reports have shown that resveratrol can reduce IL-1β-induced inflammatory signaling in osteoarthritis [21], lessen the severity of multiple sclerosis [22], and inhibit platelet aggregation in cardiovascular diseases [23]. Furthermore, resveratrol has anti-inflammatory [24], antioxidant [25], and antiaging [26] properties. Piceid (3,5,4′-trihydroxystilbene-3-*O*-β-D-glucopyranoside) is the major resveratrol derivative in many plants [27]. Piceid, like resveratrol, has potential applications in human health, including anti-inflammatory [28], antioxidative [29], and anticarcinogenic [30] applications.

Resveratrol-enriched rice was generated through genetic engineering by transferring the *Arachis hypogaea stilbene synthase* (*STS*), a resveratrol biosynthesis gene, to Dongjin (DJ) rice [31]. Rodboon et al. [32] demonstrated that germinated riceberry rice exhibited the higher antioxidations and tyrosinase-related melanogenic inhibition effects than the ungerminated riceberry rice. Cho and Lim [33] reported that the residual phenolic acid content in brown rice increased via germination and significantly increased the antioxidant activities when comparison with nongermination brown rice. Therefore, the present study aimed to investigate the effect of germination on the change in resveratrol and piceid contents in DJ526 seeds, as well as the anti-inflammatory activities of germinated and non-germinated DJ526 seed extracts.

## 2. Results

### 2.1. The Quantity of Resveratrol Content in Rice Grains

**Figure 1:**
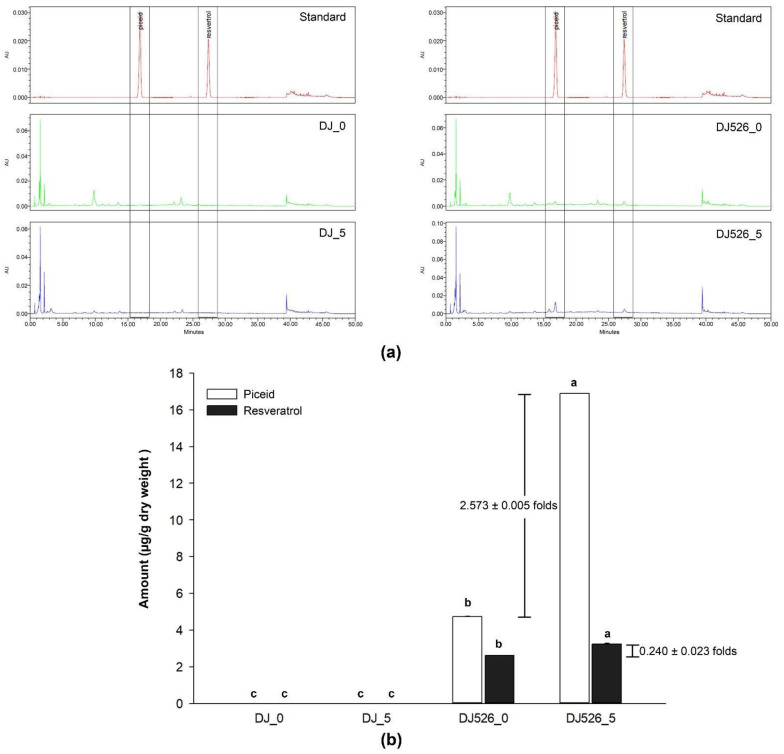
Piceid and resveratrol amounts in rice grains. (**a**) High-performance liquid chromatography (HPLC) electropherograms and (**b**) piceid and resveratrol contents calculated from the standard calibration curve. The results are presented as means ± standard deviations (*n* = 3). Lowercase letters (a–c) indicate significant differences of piceid and resveratrol contents in DJ_0, DJ_5, DJ526_0, and DJ526_5 with a *p*-value less than 0.05.

The resveratrol and piceid contents were determined using high-performance liquid chromatography (HPLC) and compared to a calibration standard mixture of resveratrol and piceid. The piceid signal peaked at a retention time of 16.947 min, while the resveratrol signal peaked at a retention time of 27.462 min. Figure 1a shows that the peaks at retention times of 16.947 and 27.462 min were not detected in the chromatograms of the normal rice seed extracts (both DJ_0 and DJ_5). However, piceid and resveratrol peaks were observed in the chromatograms of DJ526_0 and DJ526_5. The piceid and resveratrol amounts in DJ526_0 were 4.724 ± 0.02 and 2.605 ± 0.001 µg/g dry weight (dw), respectively (Figure 1b). However, the piceid and resveratrol amounts in DJ526_5 seeds (5-day-old germinated seeds) were 16.879 ± 0.024 and 3.230 ± 0.060 µg/g dw, respectively. This finding indicates that piceid and resveratrol production sequentially increased during germination. The piceid and resveratrol amounts in DJ526_5 increased 2.573 ± 0.005- and 0.240 ± 0.023-fold, respectively, when compared to a non-germinated seed (DJ526_0).

### 2.2. Lipopolysaccharide (LPS)-Induced Viability and Nitric Oxide (NO) Production in RAW264.7 Cells Treated with Rice Seed Extract

**Figure 2:**
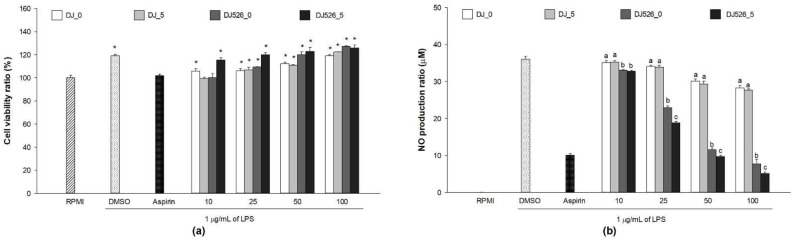
Effects of rice seed extracts on lipopolysaccharide (LPS)-stimulated RAW264.7 cells. The effect on (**a**) RAW264.7 cell viability and (**b**) nitric oxide (NO) production. The concentration of dimethyl sulfoxide (DMSO) was 0.1%, while the concentration of aspirin was 200 µg/mL. The results are presented as means ± standard deviations (*n* = 3). (*) indicates significant differences at *p*-values less than 0.05 when compared to the RPMI group. Lowercase letters (a–c) indicate significant differences of NO production with *p*-values less than 0.05 among DJ_0, DJ_5, DJ526_0, and DJ526_5 at the same concentrations.

The extract’s cytotoxicity was assessed in LPS-stimulated RAW264.7 cells by comparing the cell viability of the treated groups to that of the untreated group (RPMI). Figure 2a shows that 1 µg/mL LPS treatment significantly enhanced cell proliferation (the dimethyl sulfoxide [DMSO] group). At concentrations up to 100 µg/mL, none of the extracts were cytotoxic to LPS-stimulated RAW264.7 cells. In addition, cells treated with extracts at concentrations of 25, 50, and 100 µg/mL significantly increased proliferative activity on LPS-stimulated RAW264.7 cells when compared to the untreated group.

The inflammation environment was mocked up using the LPS model. The cells treated with 1 µg/mL LPS produced significantly more NO than the untreated group (Figure 2b). Treatment with both normal and transgenic rice seed extracts significantly reduced NO production. Furthermore, increasing the extract concentrations significantly enhanced the inhibition of LPS-induced NO production. Treatment with 10−100 µg/mL of the resveratrol-enriched rice seed extracts (DJ526_0 and DJ526_5) remarkably reduced NO production when compared to the normal rice (DJ_0 and DJ_5). However, 5-day-old germination of the resveratrol-enriched rice seed extracts (DJ526_5) significantly promoted the inhibition of LPS-induced NO production when compared to the normal transgenic rice seed extract (DJ526_0). These findings indicate that increasing the treatment concentration up to 100 µg/mL inhibited LPS-induced NO production while causing no cell cytotoxicity. Additionally, germination of resveratrol-enriched rice seeds (5 days old) declined NO production significantly when compared to non-germinated seeds.

### 2.3. mRNA Expression Levels of the Proinflammatory Genes in LPS-Stimulated RAW264.7 Cells Treated with Rice Seed Extracts

**Figure 3:**
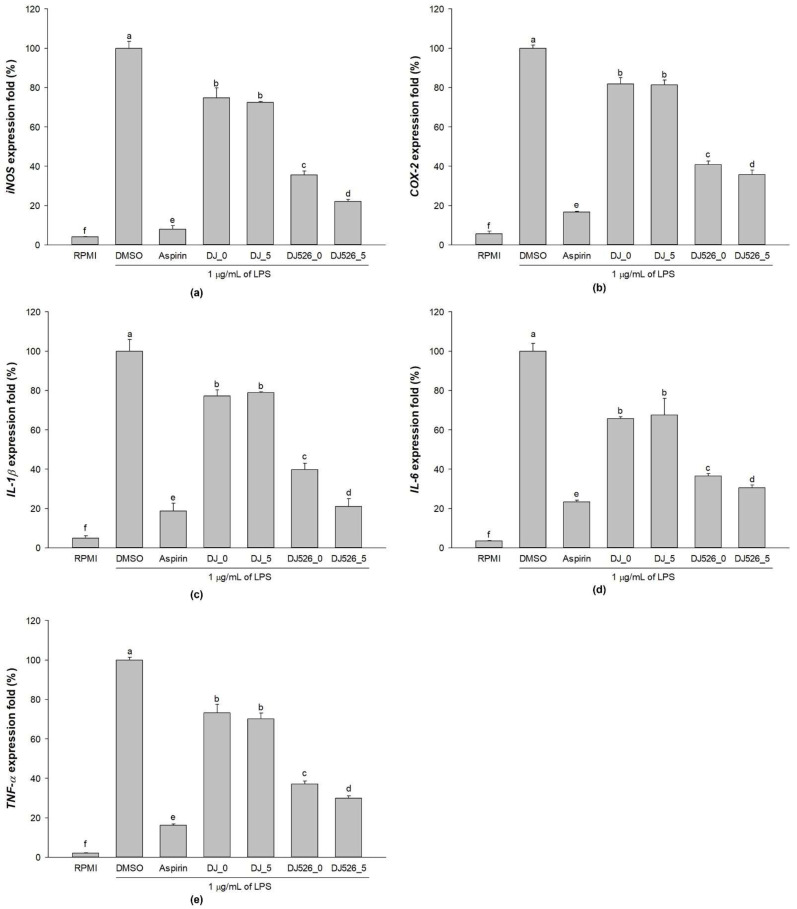
Effects of rice seed extracts on the mRNA expression levels of proinflammatory genes, including (**a**) *inducible nitric oxide synthase* (*iNOS*), (**b**) *cyclooxygenase-2* (*COX-2*), (**c**) *interleukin*(*IL*)*-1β*, (**d**) *IL-6*, and (**e**) *tumor necrosis factor-alpha* (*TNF-α*). The concentration of dimethyl sulfoxide (DMSO) was 0.1%, while the concentration of aspirin was 200 µg/mL. The results are presented as means ± standard deviations (*n* = 3). Lowercase letters (a–f) indicate significant differences of cytokine production with *p*-values less than 0.05 among DJ_0, DJ_5, DJ526_0, and DJ526_5.

The mRNA expression levels of LPS-induced proinflammatory genes were determined after six hours of LPS induction. Treatment with 1 µg/mL LPS significantly upregulated *inducible nitric oxide synthase* (*iNOS*), *cyclooxygenase-2* (*COX-2*), *IL-1β*, *IL-6*, and *TNF-α* expressions (Figure 3). Pretreatment with rice seed extracts (DJ-0, DJ_5, DJ526_0, and DJ526_5) significantly reduced LPS-induced proinflammatory gene expression levels. Compared to DJ_0, treatment with DJ_5 had no effect on proinflammatory gene expression levels, but treatment with DJ526_0 and DJ526_5 powerfully inhibited proinflammatory genes. In addition, the suppressive effect of DJ526_5 on LPS-induced proinflammatory gene expression was significantly greater than that of DJ526_0. These findings indicate that higher piceid and resveratrol amounts exerted a higher inhibitory effect on LPS-induced proinflammatory gene expression.

### 2.4. LPS-Induced Prostaglandin E2 (PGE_2_) Production in RAW264.7 Cells Treated with Rice Seed Extracts

**Figure 4:**
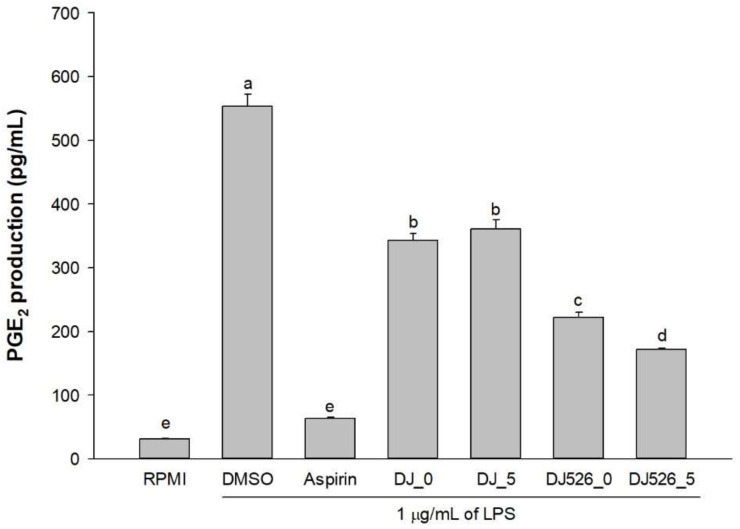
The effect of rice seed extracts on prostaglandin E2 (PGE_2_) production levels in lipopolysaccharide (LPS)-stimulated RAW264.7 cells. The concentration of dimethyl sulfoxide (DMSO) was 0.1%, while the concentration of aspirin was 200 µg/mL. The results are presented as means ± standard deviations (*n* = 3). Lowercase letters (a–e) indicate significant differences of PGE_2_ production with *p*-values less than 0.05 among DJ_0, DJ_5, DJ526_0, and DJ526_5.

PGE_2_ is a well-known inflammatory mediator [34]. Therefore, PGE_2_ production in LPS-stimulated RAW264.7 cells was investigated. Treatment with LPS significantly induced PGE_2_ production (Figure 4). However, pretreatment with rice seed extracts inhibited PGE_2_ production. There was no significant difference in PGE_2_ production between DJ_0 and DJ_5. Treatment with resveratrol-enriched rice remarkably decreased LPS-induced PGE_2_ production compared to normal rice (DJ_0 and DJ_5). Furthermore, DJ526_5-treated cells showed the highest suppression of LPS-induced PGE_2_ production. These findings indicate that increasing resveratrol and piceid amounts significantly inhibited PGE_2_ in LPS-stimulated RAW264.7 cells.

### 2.5. Inflammation-Related Pathway Activation in LPS-Stimulated RAW264.7 Cells Treated with Rice Seed Extracts

**Figure 5:**
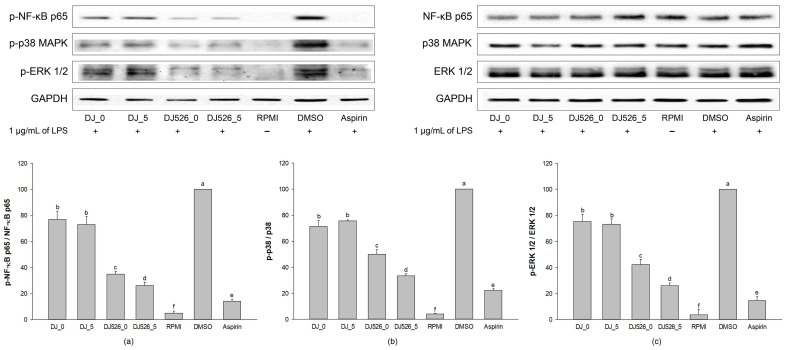
Effects of rice seed extract on inflammation-related pathway activation, including (**a**) phosphorylated nuclear factor kappa B (p-NF-κB) p65, (**b**) p-p38 mitogen-activated protein kinase (MAPK), and (**c**) phosphorylated extracellular signal-regulated kinase 1/2 (p-ERK 1/2). The concentration of dimethyl sulfoxide (DMSO) was 0.1%, while the concentration of aspirin was 200 µg/mL. The results are presented as means ± standard deviations (*n* = 2). Lowercase letters (a–f) indicate significant differences of protein expression with *p*-values less than 0.05 among DJ_0, DJ_5, DJ526_0, and DJ526_5.

Treatment with LPS (DMSO group) activated the NF-κB and MAPK signaling pathways (Figure 5) via increasing p-NF-κB p65 (Figure 5a), p-p38 MAPK (Figure 5b), and phosphorylated extracellular signal-regulated kinase 1/2 (p-ERK 1/2; Figure 5c) expression levels. Treatment with rice seed extracts significantly reduced these LPS-induced proteins. When compared to normal rice (DJ_0 and DJ_5), transgenic rice seed extracts remarkably decreased p-NF-κB p65, p-p38 MAPK, and p-ERK 1/2 levels. In addition, DJ526_5-treated cells powerfully downregulated p-NF-κB p65, p-p38 MAPK, and p-ERK 1/2 expression levels when compared to DJ526_0-treated cells. These findings indicate that treatment with rice seed extracts potentially suppressed the NF-κB and MAPK signaling pathways. In particular, the inhibition level increased as the piceid and resveratrol amounts in the transgenic rice seeds increased.

## 3. Discussion

The piceid and resveratrol contents of rice seeds were measured using HPLC. The chromatograms revealed that the retention time peaks at 16.947 (piceid) and 27.462 (resveratrol) minutes can only be detected in transgenic rice seeds, indicating that piceid and resveratrol cannot be found in normal rice seeds. For transgenic rice, DJ526_0 contained 4.727 ± 0.023 µg/g dw of piceid and 2.605 ± 0.001 µg/g dw of resveratrol, while the piceid and resveratrol contents in DJ526_5 were 16.879 ± 0.024 and 3.230 ± 0.06 µg/g dw, respectively. Piceid amounts were 1.813 ± 0.009 times higher than resveratrol amounts in DJ526_0, and 5.225 ± 0.007 times higher in DJ526_5. Our findings are consistent with those of Kuo et al. [27] and Su et al. [35], who found that piceid amounts are usually much higher than resveratrol amounts. Furthermore, Kuo et al. [36] reported that piceid amounts are 2.82-fold higher than resveratrol amounts in the root of *Polygonum cuspidatum*. The piceid and resveratrol contents tend to increase in germinated DJ526 (DJ526_5) when compared to nongerminated DJ526. Similarly, Hung and Chen [37] suggested that the amounts of resveratrol and γ-aminobutyric acid increased with germination time. Wang et al. [38] demonstrated that the resveratrol content in the whole sprout of peanuts increased during germination. On Day 9, the resveratrol contents in the whole sprout of Tainan Selected 9 (TNS 9), Tainan 11 (TN 11), and Tainan 14 (TN 14) were 5.38-, 5.71-, and 5.09-fold higher than those on Day 0, respectively.

Macrophages can be activated by LPS via multiple signaling pathways, including NF-κB and MAPK [39,40,41]; upregulating proinflammatory cytokines, such as IL-1β, IL-6, and TNF-α; and inflammatory mediators, such as NO, PGE_2_, and iNOS [20,42]. Therefore, LPS is often used to mimic the inflammatory environment in macrophages [43,44,45]. Our findings demonstrated that resveratrol-enriched rice inhibited NF-κB and MAPK signaling pathways by lowering NF-κB p65, ERK 1/2, and p38 MAPK phosphorylation. This reduced the LPS-induced NO production (Figure 2b), PGE_2_ production (Figure 4), and proinflammatory genes (*iNOS*, *COX-2*, *IL-1β*, *IL-6*, and *TNF-α*). The increase in piceid and resveratrol contents was correlated with an increase in anti-inflammatory properties. Treatment with DJ526 significantly suppressed NO production in LPS-stimulated cells. Compared to DJ526_0, treatment with DJ526_5, which contains a higher amount of piceid and resveratrol, significantly reduced NO production in a concentration-dependent manner. Furthermore, NO production was found to be significantly correlated with the amount of piceid (Pearson’s correlation, *r* = −0.710, *p* = 0.01) and resveratrol (Pearson’s correlation, *r* = −0.890, *p* = 0.01). The findings are consistent with those from various experiments that found resveratrol to have an inhibitory effect on NO production [46,47,48]. Similarly, Zong et al. [20] found that resveratrol at 1–10 µM significantly reduced ERK 1/2 and p38 MAPK phosphorylation, reducing NO and PGE_2_ production, TNF-α, and IL-1β levels, as well as iNOS and COX-2 mRNA and protein expression levels. Zimmermann-Franco et al. [49] reported the inhibition effect of resveratrol on the production of pro-inflammatory mediators such as NO, IL-1β, IL-6, and TNF-α. The anti-inflammatory potential of resveratrol was also demonstrated in an animal model. According to Simão et al. [50], resveratrol reduces neuroinflammation in rats via downregulating NF-κB-related proteins, COX-2, and iNOS. In addition, Zimmermann-Franco et al. [49] demonstrated the in vivo anti-inflammatory effect of resveratrol in a mouse model of croton-oil-induced ear edema. Furthermore, Su et al. [51] found that high-dose resveratrol (150 mg/kg body weight) reduced inflammatory responses in C57BL/6J mice. Consequently, treatment with resveratrol-enriched rice (DJ526_0 and DJ526_5) inhibited LPS-induced inflammatory responses, which correlated with resveratrol content accumulation in transgenic rice.

## 4. Materials and Methods

### 4.1. Plant Materials

DJ and DJ526 rice seeds were received from the Rural Development Administration (Jeonju, Republic of Korea). They were unpeeled and sterilized in a cleaner solution (70% [*v*/*v*] of ethanol and 5% [*v*/*v*] of hypochlorite) for one hour. The sterilized seeds were divided into two groups. The first group was ground into a fine powder and designated as Day 0 (DJ_0 and DJ526_0). The second group was allowed to germinate in autoclaved water for five days (DJ_5 and DJ526_5). The 5-day-old seeds were collected and ground into a fine powder. The samples were extracted as previously described [52], with the exception that the extraction buffer was changed from 100% methanol to 80% methanol. The extracts were prepared at concentrations of 10, 25, 50, and 100 mg/mL in DMSO and were diluted with cell culture medium at the concentrations of 10, 25, 50, and 100 µg/mL for in vitro experiments.

### 4.2. Piceid and Resveratrol Content Quantification

**Figure 6:**
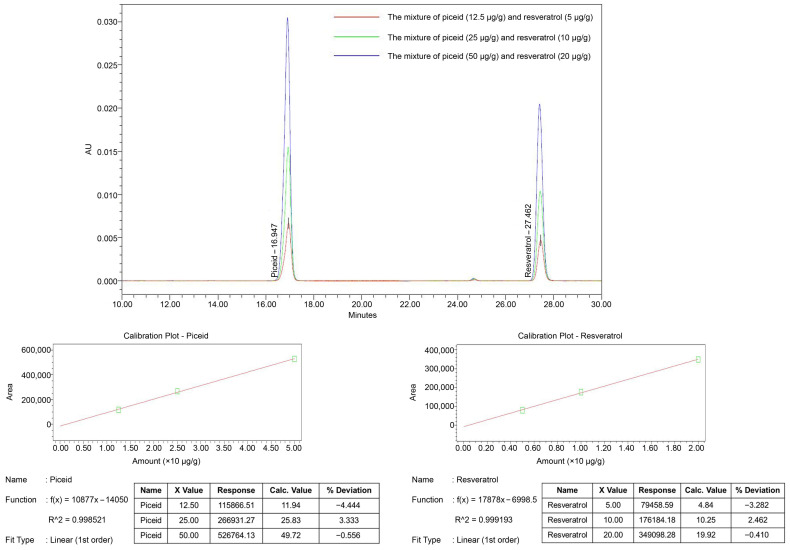
Piceid (12.5–50.0 µg/g) and resveratrol (5.0–20.0 µg/g) calibration standard curves.

To determine the piceid and resveratrol content in rice grains, a fine powder of each sample was mixed with 80% methanol (300 mg fine powder: 900 µL of 80% methanol). The mixture was sonicated for 30 min at room temperature. The tube was centrifuged at 10,000× *g* at 4 °C for 5 min. After centrifugation, the supernatant was collected and filtered through a 0.2 m nylon membrane filter. The filtered supernatant (1 µL) was used for HPLC analysis of piceid and resveratrol amounts on a Waters e2695 (Waters, Milford, MA, USA). HPLC was performed as previously described [53]. Piceid and resveratrol contents were quantified by comparing them to the calibration standard curve (Figure 6).

### 4.3. RAW264.7 Cell Viability and NO Production

RAW264.7 cells (Korean Cell Line Bank, Seoul, Republic of Korea) were seeded in a 96-well plate at a concentration of 1 × 10^5^ cells/well. The plate was maintained in an environment-controlled incubator (37 °C and 5% CO_2_) for 24 h. The culture medium was replaced with various concentrations of rice seed extracts or 200 µg/mL of aspirin (positive control) [54,55] prepared in the nonphenol red Roswell Park Memorial Institute 1640 medium. After one hour of incubation, cells were stimulated with or without 1 µg/mL LPS. The plate was incubated at 37 °C with 5% CO_2_ for another 24 h. The culture medium (100 µL) was transferred to a new 96-well plate. The same volume of Griess reagent (100 µL; Sigma-Aldrich, St. Louis, MO, USA) was added to each well, and the plate was incubated at room temperature (light-protected) for 15 min. The NO production was evaluated by measuring the absorbance at 540 nm and quantified using a standard curve of sodium nitrite [NaNO_2_; Figure S1; (1)]. For the original plate, an EZ-Cytox Cell Viability Assay Kit (10 µL; DoGenBio, Seoul, Republic of Korea) was added to each well. The plate was incubated at 37 °C for four hours. The cell viability was calculated according to the following Formula (2).

(1) $$\mathrm{NO}\;\mathrm{production}\;\left(µM\right)=91.801x\;-\;5.2398,$$

where “x” represents the absorbance value at 540 nm.

(2) $$\mathrm{Cell}\;\mathrm{viability}\;\mathrm{ratio}\;\left(\%\right)=\frac{\mathrm{Absorbance}\;\mathrm{at}\;450\;\mathrm{nm}\;\mathrm{for}\;\mathrm{the}\;\mathrm{treatment}}{\mathrm{Absorbance}\;\mathrm{at}\;450\;\mathrm{nm}\;\mathrm{for}\;\mathrm{the}\;\mathrm{control}}\;\times \;100$$

where “control” represents the nontreatment group.

### 4.4. RNA Extraction and cDNA Synthesis

RAW264.7 cells were seeded in a 24-well plate (5 × 10^5^ cells/well) and incubated at 37 °C with 5% CO_2_ for 24 h. The culture medium was replaced with 100 µg/mL of each treatment. After one hour of incubation, LPS was added to each well to achieve a final concentration of 1 µg/mL, except for the untreated group (RPMI). The treated cells were harvested after six hours of LPS stimulation. They were washed twice with ice-cold 1× phosphate-buffered saline. The total RNA was extracted using Tri reagent™ (Invitrogen, Waltham, MA, USA) at room temperature and precipitated using 100% isopropanol at 4 °C. The RNA pellet was washed in 7% ethanol. The total RNA was quantified and qualified using a SpectraMax^®^ ABS Plus Microplate Reader (Molecular Devices, San Jose, CA, USA). The extracted RNA (1000 ng) was transcribed into cDNA using a Power cDNA Synthesis Kit (Intron Biotechnology, Seongnam-si, Republic of Korea).

### 4.5. mRNA Expression Level Measurement of the Proinflammatory Genes Using Real-Time Polymerase Chain Reaction (PCR)

RealMOD™ Green W^2^ 2 × qPCR Mix (Intron Biotechnology, Seongnam-si, Republic of Korea) and the CFX Connect Real-Time PCR System (Bio-Rad, Hercules, CA, USA) were used to measure the mRNA expression levels of proinflammatory genes (*IL-1β*, *IL-6*, *TNF-α*, *iNOS*, *COX-2*, and *β-actin*). The PCR reaction consisted of 0.375 M of each primer (Table 1) and 5 ng of cDNA template. The PCR condition was conducted as previously described [52]. The gene expression levels (fold changes) were analyzed using the CFX Maestro software (accessed on: 19 May 2023), with *β-actin* serving as a reference gene.

### 4.6. PGE_2_ Production Measurement

The supernatant of the culture medium was collected from each treatment in a 1.5 mL microtube. The tubes were centrifuged at 3000 rpm for 10 min at room temperature. PGE_2_ production was measured using a PGE_2_ enzyme-linked immunosorbent assay kit (ADI900-001: Enzo Life Sciences, Farmingdale, NY, USA) according to the manufacturer’s instructions. The PGE_2_ production was calculated using a standard curve [Figure S2; (3)].

(3) $${\mathrm{PGE}}_{2}\;\mathrm{production}\;\left(\mathrm{pg}/\mathrm{mL}\right)=130,516x^{-1.729},$$

where “x” represents the percent-bound value.

### 4.7. Western Blot Analysis

The treated cells were collected in a 1.5 mL microtube and lysed on ice for 30 min in the lysis buffer (radioimmunoprecipitation assay buffer; Geneall Biotechnology, Seoul, Republic of Korea) supplemented with 1× Protease Inhibitor Cocktail Kit 5 (Bio-Medical Science Co., Ltd., Seoul, Republic of Korea). The tubes were centrifuged at 13,000 rpm and 4 °C for 30 min. The supernatant was transferred to the new microtubes. The protein concentration was measured using the Bradford reagent (Sigma-Aldrich, St. Louis, MO, USA). Proteins from each treatment (30 µg) were separated using 10% sodium dodecyl-sulfate polyacrylamide gel electrophoresis. The separated proteins were transferred onto a nitrocellulose membrane. The primary antibodies specific to p-NF-κB p65 (Cell Signaling, Danvers, MA, USA), NF-κB p65 (Santa Cruz Biotechnology, Dallas, TX, USA), p-p38 MAPK (Cell Signaling, Danvers, MA, USA), p38 MAPK (Santa Cruz Biotechnology, Dallas, TX, USA), p-ERK 1/2 (Cell Signaling, Danvers, MA, USA), ERK 1/2 (Cell Signaling, Danvers, MA, USA), and glyceraldehyde-3-phosphate dehydrogenase (Santa Cruz Biotechnology, Dallas, TX, USA) were applied and incubated at 4 °C for overnight. After that, the secondary antibodies, goat antirabbit IgG (H + L)-horseradish peroxidase (GenDEPOT, Barker, TX, USA) or m-IgGκ BP-horseradish peroxidase (Santa Cruz Biotechnology, Dallas, TX, USA), were applied onto the membrane and incubated at room temperature for one hour. Protein signaling was detected using Clarity™ Western ECL Substrate (Bio-Rad, Hercules, CA, USA), and the detected signals were imaged and quantified in terms of intensity using a ChemiDoc Imaging system (Bio-Rad, Hercules, CA, USA).

### 4.8. Statistical Analysis

The data were expressed as means and standard deviations. All statistical analyses were performed using Statistix (version 8.1; Statistix, Tallahassee, FL, USA) (accessed on 19 May 2023). The data were analyzed using a one-way analysis of variance, followed by post hoc Duncan’s multiple range tests. The two groups were compared using Student’s *t*-test (*p* < 0.05).

## 5. Conclusions

In this study, the anti-inflammatory effects of resveratrol-enriched rice (DJ526_0 and DJ526_5) were investigated in LPS-stimulated RAW264.7 cells. The NF-κB and MAPK pathways were inactivated in the DJ526_0- and DJ526_5-treated cells via decreasing p-NF-κB p65, p-ERK 1/2, and p-p38 MAPK, suppressing NO and PGE_2_ production, as well as proinflammatory gene expression levels. Furthermore, piceid and resveratrol contents can rise during germination and significantly enhance anti-inflammatory activities in LPS-stimulated RAW264.7 cells. We suggest that resveratrol-enriched rice could be developed and used as an anti-inflammatory agent following further research.

## Figures and Tables

**Table 1:** Sequences of the primers used in this study.

Gene	Accession No.	Sequences (5′–3′)	Target Size (bp)
*IL-1β*	NM_008361.4	Forward: GGG CCT CAA AGG AAA GAA TC Reverse: TAC CAG TTG GGG AAC TCT GC	183
*IL-6*	NM_031168.2	Forward: AGT TGC CTT CTT GGG ACT GA Reverse: CAG AAT TGC CAT TGC ACA AC	191
*COX-2*	NM_011198.4	Forward: AGA AGG AAA TGG CTG CAG AA Reverse: GCT CGG CTT CCA GTA TTG AG	194
*iNOS*	BC062378.1	Forward: TTC CAG AAT CCC TGG ACA AG Reverse: TGG TCA AAC TCT TGG GGT TC	180
*TNF-α*	D84199.2	Forward: ATG AGC ACA GAA AGC ATG ATC Reverse: TAC AGG CTT GTC ACT CGA ATT	276
*β-actin*	NM_007393.5	Forward: CCA CAG CTG AGA GGA AAT C Reverse: AAG GAA GGC TGG AAA AGA GC	193

## Data Availability

All applicable data have been provided in the manuscript. The authors will provide additional details if necessary.

## Supplementary Materials

The following supporting information can be downloaded at: https://www.mdpi.com/article/10.3390/molecules28134898/s1, Figure S1: Standard curve of sodium nitrite over the concentration range of 0–100 µM, Figure S2: Standard curve of prostaglandin E2 (PGE_2_) over the concentration range of 39.0625–2500 pg/mL.

## Sample Availability

Samples of the compounds DJ_0, DJ_5, DJ526_0, and DJ526_5 are available from the authors.

## References

[1] Nathan C. (2002). Points of control in inflammation. Nature.

[2] Lee E., Shin S.-Y., Kim J.-K., Woo E.-R., Kim Y.-M. (2012). Anti-inflammatory effects of amentoflavone on modulation of signal pathways in LPS-stimulated RAW264.7 cells. Bull. Korean Chem. Soc..

[3] Zhang W., Yan J., Wu L., Yu Y., Ye R.D., Zhang Y., Liang X. (2019). In vitro immunomodulatory effects of human milk oligosaccharides on murine macrophage RAW264.7 cells. Carbohydr. Polym..

[4] Dinarello C.A. (1997). Proinflammatory and anti-inflammatory cytokines as mediators in the pathogenesis of septic shock. Chest.

[5] Nathan C., Shiloh Michael U. (2000). Reactive oxygen and nitrogen intermediates in the relationship between mammalian hosts and microbial pathogens. Proc. Natl. Acad. Sci. USA.

[6] Romano M., Sironi M., Toniatti C., Polentarutti N., Fruscella P., Ghezzi P., Faggioni R., Luini W., van Hinsbergh V., Sozzani S. (1997). Role of IL-6 and its soluble receptor in induction of chemokines and leukocyte recruitment. Immunity.

[7] Kishimoto T. (2010). IL-6: From its discovery to clinical applications. Int. Immunol..

[8] Gopinath V.K., Musa M., Samsudin A.R., Sosroseno W. (2006). Role of interleukin-1beta and tumour necrosis factor-alpha on hydroxyapatite-induced phagocytosis by murine macrophages (RAW264.7 cells). Br. J. Biomed. Sci..

[9] Yencilek F., Yildirim A., Yilmaz S.G., Altinkilic E.M., Dalan A.B., Bastug Y., Isbir T. (2015). Investigation of interleukin-1β polymorphisms in prostate cancer. Anticancer Res..

[10] Chen X.W., Zhou S.F. (2015). Inflammation, cytokines, the IL-17/IL-6/STAT3/NF-κB axis, and tumorigenesis. Drug. Des. Dev. Ther..

[11] Conway E.M., Pikor L.A., Kung S.H., Hamilton M.J., Lam S., Lam W.L., Bennewith K.L. (2016). Macrophages, Inflammation, and Lung Cancer. Am. J. Respir. Crit. Care Med..

[12] Donath M.Y., Shoelson S.E. (2011). Type 2 diabetes as an inflammatory disease. Nat. Rev. Immunol..

[13] Thao N.P., Cuong N.X., Luyen B.T.T., Quang T.H., Hanh T.T.H., Kim S., Koh Y.-S., Nam N.H., Kiem P.V., Minh C.V. (2013). Anti-inflammatory components of the starfish *Astropecten polyacanthus*. Mar. Drugs.

[14] Djoko B., Chiou R.Y.Y., Shee J.-J., Liu Y.-W. (2007). Characterization of immunological activities of peanut stilbenoids, arachidin-1, piceatannol, and resveratrol on lipopolysaccharide-induced inflammation of RAW 264.7 macrophages. J. Agric. Food Chem..

[15] Li Z., Chen X., Liu G., Li J., Zhang J., Cao Y., Miao J. (2021). Antioxidant activity and mechanism of resveratrol and polydatin isolated from mulberry (*Morus alba* L.). Molecules.

[16] Singh C.K., Liu X., Ahmad N. (2015). Resveratrol, in its natural combination in whole grape, for health promotion and disease management. Ann. N. Y. Acad. Sci..

[17] Vitaglione P., Sforza S., Galaverna G., Ghidini C., Caporaso N., Vescovi P.P., Fogliano V., Marchelli R. (2005). Bioavailability of trans-resveratrol from red wine in humans. Mol. Nutr. Food Res..

[18] Chen B.-Y., Kuo C.-H., Liu Y.-C., Ye L.-Y., Chen J.-H., Shieh C.-J. (2012). Ultrasonic-assisted extraction of the botanical dietary supplement resveratrol and other constituents of *Polygonum cuspidatum*. J. Nat. Prod..

[19] Shakibaei M., Harikumar K.B., Aggarwal B.B. (2009). Resveratrol addiction: To die or not to die. Mol. Nutr. Food Res..

[20] Zong Y., Sun L., Liu B., Deng Y.S., Zhan D., Chen Y.L., He Y., Liu J., Zhang Z.J., Sun J. (2012). Resveratrol inhibits LPS-induced MAPKs activation via activation of the phosphatidylinositol 3-kinase pathway in murine RAW 264.7 macrophage cells. PLoS ONE.

[21] Shakibaei M., Csaki C., Nebrich S., Mobasheri A. (2008). Resveratrol suppresses interleukin-1β-induced inflammatory signaling and apoptosis in human articular chondrocytes: Potential for use as a novel nutraceutical for the treatment of osteoarthritis. Biochem. Pharmacol..

[22] Imler T.J., Petro T.M. (2009). Decreased severity of experimental autoimmune encephalomyelitis during resveratrol administration is associated with increased IL-17+IL-10+ T cells, CD4− IFN-γ+ cells, and decreased macrophage IL-6 expression. Int. Immunopharmacol..

[23] Brookins Danz E.D., Skramsted J., Henry N., Bennett J.A., Keller R.S. (2009). Resveratrol prevents doxorubicin cardiotoxicity through mitochondrial stabilization and the Sirt1 pathway. Free Radic. Biol. Med..

[24] Nunes S., Danesi F., Del Rio D., Silva P. (2018). Resveratrol and inflammatory bowel disease: The evidence so far. Nutr. Res. Rev..

[25] Meng Q., Guo T., Li G., Sun S., He S., Cheng B., Shi B., Shan A. (2018). Dietary resveratrol improves antioxidant status of sows and piglets and regulates antioxidant gene expression in placenta by Keap1-Nrf2 pathway and Sirt1. J. Anim. Sci. Biotechnol..

[26] Alarcón de la Lastra C., Villegas I. (2005). Resveratrol as an anti-inflammatory and anti-aging agent: Mechanisms and clinical implications. Mol. Nutr. Food Res..

[27] Kuo C.-H., Chen B.-Y., Liu Y.-C., Chen J.-H., Shieh C.-J. (2016). Production of resveratrol by piceid deglycosylation using cellulase. Catalysts.

[28] Lanzilli G., Cottarelli A., Nicotera G., Guida S., Ravagnan G., Fuggetta M.P. (2012). Anti-inflammatory effect of resveratrol and polydatin by in vitro IL-17 modulation. Inflammation.

[29] Bröhan M., Jerkovic V., Collin S. (2011). Potentiality of red sorghum for producing stilbenoid-enriched beers with high antioxidant activity. J. Agric. Food Chem..

[30] Soleas G.J., Goldberg D.M., Grass L., Levesque M., Diamandis E.P. (2001). Do wine polyphenols modulate p53 gene expression in human cancer cell lines?. Clin. Biochem..

[31] Baek S.-H., Shin W.-C., Ryu H.-S., Lee D.-W., Moon E., Seo C.-S., Hwang E., Lee H.-S., Ahn M.-H., Jeon Y. (2013). Creation of resveratrol-enriched rice for the treatment of metabolic syndrome and related diseases. PLoS ONE.

[32] Rodboon T., Okada S., Suwannalert P. (2020). Germinated riceberry rice enhanced protocatechuic acid and vanillic acid to suppress melanogenesis through cellular oxidant-related tyrosinase activity in B16 cells. Antioxidants.

[33] Cho D.H., Lim S.T. (2018). Changes in phenolic acid composition and associated enzyme activity in shoot and kernel fractions of brown rice during germination. Food Chem..

[34] Gomez I., Foudi N., Longrois D., Norel X. (2013). The role of prostaglandin E2 in human vascular inflammation. Prostagland. Leukot. Essent. Fat. Acids.

[35] Su D., Cheng Y., Liu M., Liu D., Cui H., Zhang B., Zhou S., Yang T., Mei Q. (2013). Comparision of piceid and resveratrol in antioxidation and antiproliferation activities in vitro. PLoS ONE.

[36] Kuo C.H., Chen B.Y., Liu Y.C., Chang C.M., Deng T.S., Chen J.H., Shieh C.J. (2013). Optimized ultrasound-assisted extraction of phenolic compounds from *Polygonum cuspidatum*. Molecules.

[37] Hung C.-H., Chen S.-D. (2022). Study of inducing factors on resveratrol and antioxidant content in germinated peanuts. Molecules.

[38] Wang K.-H., Lai Y.-H., Chang J.-C., Ko T.-F., Shyu S.-L., Chiou R.Y.Y. (2005). Germination of peanut kernels to enhance resveratrol biosynthesis and prepare sprouts as a functional vegetable. J. Agric. Food Chem..

[39] Lu Y.-C., Yeh W.-C., Ohashi P.S. (2008). LPS/TLR4 signal transduction pathway. Cytokine.

[40] Grylls A., Seidler K., Neil J. (2021). Link between microbiota and hypertension: Focus on LPS/TLR4 pathway in endothelial dysfunction and vascular inflammation, and therapeutic implication of probiotics. Biomed. Pharmacother..

[41] Ciesielska A., Matyjek M., Kwiatkowska K. (2021). TLR4 and CD14 trafficking and its influence on LPS-induced pro-inflammatory signaling. Cell. Mol. Life Sci..

[42] Arthur J.S., Ley S.C. (2013). Mitogen-activated protein kinases in innate immunity. Nat. Rev. Immunol..

[43] Cheng D., Zhu C., Liang Y., Xing Y., Shi C. (2020). MiR-424 overexpression protects alveolar epithelial cells from LPS-induced apoptosis and inflammation by targeting FGF2 via the NF-κB pathway. Life Sci..

[44] Wang Y., Cui X.-L., Liu Y.-F., Gao F., Wei D., Li X.-W., Wang H.-N., Tan Q.-R., Jiang W. (2011). LPS inhibits the effects of fluoxetine on depression-like behavior and hippocampal neurogenesis in rats. Prog. Neuropsychopharmacol. Biol. Psychiatry.

[45] Ostos M.A., Recalde D., Zakin M.M., Scott-Algara D. (2002). Implication of natural killer T cells in atherosclerosis development during a LPS-induced chronic inflammation. FEBS Lett..

[46] Qureshi A.A., Guan X.Q., Reis J.C., Papasian C.J., Jabre S., Morrison D.C., Qureshi N. (2012). Inhibition of nitric oxide and inflammatory cytokines in LPS-stimulated murine macrophages by resveratrol, a potent proteasome inhibitor. Lipids Health Dis..

[47] Kimbrough C.W., Lakshmanan J., Matheson P.J., Woeste M., Gentile A., Benns M.V., Zhang B., Smith J.W., Harbrecht B.G. (2015). Resveratrol decreases nitric oxide production by hepatocytes during inflammation. Surgery.

[48] DiNatale J.C., Crowe-White K.M. (2022). Effects of resveratrol supplementation on nitric oxide-mediated vascular outcomes in hypertension: A systematic review. Nitric Oxide.

[49] Zimmermann-Franco D.C., Esteves B., Lacerda L.M., Souza I.d.O., Santos J.A.d., Pinto N.d.C.C., Scio E., da Silva A.D., Macedo G.C. (2018). In vitro and in vivo anti-inflammatory properties of imine resveratrol analogues. Bioorg. Med. Chem..

[50] Simão F., Matté A., Pagnussat A.S., Netto C.A., Salbego C.G. (2012). Resveratrol preconditioning modulates inflammatory response in the rat hippocampus following global cerebral ischemia. Neurochem. Int..

[51] Su L.-Y., Huang W.-C., Kan N.-W., Tung T.-H., Huynh L.B., Huang S.-Y. (2023). Effects of resveratrol on muscle inflammation, energy utilisation, and exercise performance in an eccentric contraction exercise mouse model. Nutrients.

[52] Monmai C., Kim J.-S., Baek S.-H. (2022). Transgenic rice seed extracts exert immunomodulatory effects by modulating immune-related biomarkers in RAW264.7 macrophage cells. Nutrients.

[53] Kantayos V., Shin W.-C., Kim J.-S., Jeon S.-H., Rha E.-S., Baek S.-H. (2021). Resveratrol-enriched rice identical to original Dongjin rice variety with respect to major agronomic traits in different cultivation years and regions. GM Crops Food.

[54] Liu Y., Fang S., Li X., Feng J., Du J., Guo L., Su Y., Zhou J., Ding G., Bai Y. (2017). Aspirin inhibits LPS-induced macrophage activation via the NF-κB pathway. Sci. Rep..

[55] Wang Q., Liu W., Yue Y., Sun C., Zhang Q. (2020). Proteoglycan from *Bacillus* sp. BS11 inhibits the inflammatory response by suppressing the MAPK and NF-κB pathways in lipopolysaccharide-induced RAW264.7 macrophages. Mar. Drugs.

