# Supplementary material for: Use of Germination to Enhance Resveratrol Content and Its Anti-Inflammatory Activity in Lipopolysaccharide-Stimulated RAW264.7 Cells
Source: Molecules. 2023 Jun 21;28(13):4898. doi: 10.3390/molecules28134898 (PMC10343481; doi:10.3390/molecules28134898)
Supplement: Supplementary file 1 [file molecules-28-04898-s001.zip › molecules-2433274-supplementary.pdf]

Supplementary Materials

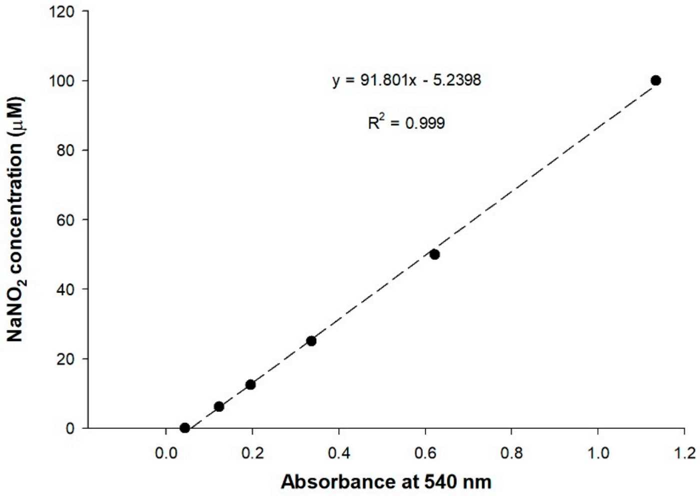

$NaNO_2$ concentration (μM)	Mean Absorbance
100	1.134
50	0.622
25	0.336
12.5	0.195
6.25	0.122
0	0.044

Figure S1. Standard curve of sodium nitrite over the concentration range of 0 – 100 μM

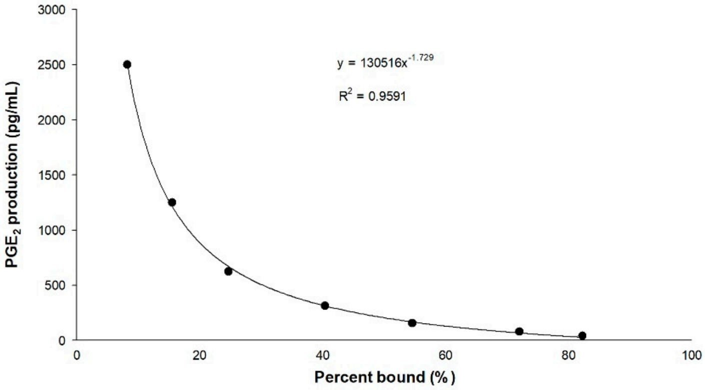

Sample	Mean OD (– Blank)	Average Net OD	Percent Bound	Concentration (pg/mL)
Blank OD	0.090			
TA	1.116			
NSB	0.003	0.000		
B0	0.623	0.620	100.00	0
Standard 1	0.054	0.051	8.23	2500
Standard 2	0.099	0.096	15.50	1250
Standard 3	0.156	0.153	24.70	625
Standard 4	0.253	0.250	40.36	312.5
Standard 5	0.341	0.338	54.56	156.25
Standard 6	0.449	0.446	71.99	78.125
Standard 7	0.513	0.510	82.24	39.0625

Figure S2. Standard curve of PGE_2 amount over the concentration range of 39.0625 – 2500 pg/mL
